# Quorum Sensing and Spoilage Potential of Psychrotrophic Enterobacteriaceae Isolated from Milk

**DOI:** 10.1155/2018/2723157

**Published:** 2018-10-22

**Authors:** Maurilio Lopes Martins, Uelinton Manoel Pinto, Katharina Riedel, Maria Cristina Dantas Vanetti

**Affiliations:** ^1^Department of Food Science and Technology, Federal Institute of Southeast of Minas Gerais, 36180-000 Rio Pomba, MG, Brazil; ^2^Food Research Center, Department of Food and Experimental Nutrition, Faculty of Pharmaceutical Sciences, University of São Paulo, 05508-000 São Paulo, SP, Brazil; ^3^Institute of Microbiology, Ernst-Moritz-Arndt University of Greifswald, 17489, Germany; ^4^Department of Microbiology, Federal University of Viçosa, 36570-000 Viçosa, MG, Brazil

## Abstract

The 16S rDNA of six psychrotrophic Enterobacteriaceae isolated from cold raw milk were sequenced and the isolate 039 was identified as* Pantoea* sp., isolates 059, 068, and 071 were identified as* Hafnia alvei*, 067 was identified as* Enterobacter* sp., and 099 was identified as* Aeromonas hydrophila*. They presented different spoilage potentials in milk with* A. hydrophila* 099 being the most deteriorative. Only* Pantoea* sp. 039 was not able to induce the quorum sensing monitor strains of acyl homoserine lactones (AHLs). The* halI* gene, which encodes the AHL synthase in* H. alvei*, was identified in the isolates 059, 067, 068, and 071. After initial sequencing characterization and cloning, this gene showed its function by the heterologous synthesis of N-hexanoyl-DL-homoserine lactone and N-3-oxohexanoyl-L-homoserine lactone in* Escherichia coli*. In addition to producing AHLs,* A. hydrophila *099 produced AI-2 in higher level than the assay's positive control* Vibrio harveyi *BB120. Therefore, Enterobacteriaceae strains isolated from cooled raw milk produce a rich array of signaling molecules that may influence bacterial traits in the milk environment.

## 1. Introduction

Contamination of products with psychrotrophic microorganisms is a concern for the dairy industry since the majority of these products are maintained and distributed at temperatures permissive for the growth of these organisms. The diverse microorganisms categorized as psychrotrophic are ubiquitous in nature and can be isolated from soil, water, and vegetation [1].

Gram-negative bacteria usually account for more than 90% of the microbial population in cold raw milk that has been stored [2] and is composed mainly of psychrotrophic species of* Pseudomonas*,* Achromobacter*,* Aeromonas*,* Serratia*,* Alcaligenes*,* Hafnia*,* Chromobacterium*,* Flavobacterium,* and* Enterobacter* [3]. Most of these bacteria produce extracellular proteolytic and lipolytic enzymes that are secreted into milk and many of them are not completely inactivated by pasteurization or by ultrahigh temperature treatment [2]. The residual activities of these enzymes can reduce the sensorial quality and shelf life of processed milk products [4].

Many bacteria regulate the expression of some genes in response to the population size in a mechanism known as quorum sensing [5]. This mechanism allows the cells to control many of their functions and depending on the signal concentration the target genes are either activated or repressed [6].

Gram [7] has shown that some strains of Enterobacteriaceae isolated from foods produce acyl homoserine lactones (AHLs). These signaling molecules were detectable from naturally contaminated foods and from samples to which pure cultures have been added when levels of Enterobacteriaceae reached 10^5^ to 10^7^ CFU/g. These levels are not uncommon in foods, which indicate that AHLs could be implicated in regulating phenotypes important for food spoilage. Additionally, it is important to understand the behavior of Enterobacteriaceae in foods since many foodborne pathogens belong to this family.

According to Christensen [8], several hydrolytic enzymes produced by a typical member of food spoilage microbiota are regulated by quorum sensing. They showed that quorum sensing is involved in the production of spoilage characteristics* in situ* on food products. AHL-production seems to be common among proteolytic psychrotrophic bacteria isolated from raw milk and the possibility of cross-communication between these psychrotrophic microbiota types was suggested [9].

The understanding of the role that quorum sensing plays in the regulation of spoilage phenotypes in bacteria from milk is relevant and may be used to create new strategies to preserve dairy products. Therefore, the purpose of the present work was to elucidate which signaling molecules are produced by proteolytic psychrotrophic Enterobacteriaceae bacteria isolated from cooled raw milk and to relate the quorum sensing mechanism to the spoilage potential of these strains.

## 2. Material and Methods

### 2.1. Strains and Growth Conditions

The psychrotrophic strains and other bacteria used in the present study are listed in Table 1. Unless otherwise stated, these strains were grown at 30°C in Luria-Bertani (LB) medium. Solid media were routinely solidified with 1.4% agar. Antibiotics were added as required at final concentrations of 20 *μ*g/mL for gentamicin and tetracycline, 50 *μ*g/mL for spectinomycin, and 100 *μ*g/mL for ampicillin.

### 2.2. Identification of Psychrotrophic Strains Isolated from Cooled Raw Milk

Psychrotrophic bacteria were isolated from cooled raw milk as described by Martins [10] and in order to confirm their identity we have used API ID32E (BioMérieux, Marcy-l'Etoile, France) for phenotypic characterization and 16S rDNA sequencing for genotypic characterization.

For sequencing the rDNA 16S, PCR reaction consisted of 25 mM MgCl_2_, 5.0 *μ*L of 10X buffer Ex* Taq*, 25 mM deoxynucleotide triphosphates (dNTPs), 25 *μ*M of each primer, 1 U Ex* Taq* DNA polymerase, and 40 ng of DNA in a final volume of 50 *μ*L. Primers described by Juretschko [11] are as follows: 616V, 5'AGAGTTTGATYMTGGCTC3', and 630R, 5'CAKAAAGGAGGTGATCC3', were synthesized by Microsynth (Zürich, Switzerland). PCR reactions were carried out in a T3 thermocycler (Biometra®, Biolabo Scientific Instruments, Zürich, Switzerland). The M13 Universal and Reverse Primers were used to sequence the rDNA 16S genes cloned into pCR2.1-TOPO. Thereafter, the obtained sequences were used to search for similarity using the Ribosomal Database Project II (http://rdp.cme.msu.edu/seqmatch/seqmatch_result.jsp?qvector=204&depth=0&currentRoot=419&num=20).

### 2.3. Food Spoilage Potential and Production of Exoenzymes by Psychrotrophic Strains

To evaluate the food spoilage potential, samples of reconstituted skim milk powder, 12% (w/v), were inoculated with approximately 1.0 x 10^4^ CFU/mL of* Pantoea *sp. 039,* H. alvei* 059, 068, or 071,* Enterobacter* sp. 067, or* A. hydrophila* 099. The samples were incubated at room temperature for 18 h and thereafter their spoilage ability in milk was checked visually.

Proteolytic activity was also determined by streaking the strains onto LB agar plates supplemented with 2% (w/v) skim milk powder and on azocasein as previously prepared [4]. Prior to enzymatic assays, the method of Bradford [12] was used for quantitative protein determination using bovine serum albumin (BSA) as a standard.

Supernatant proteins obtained as crude extracts by centrifuging cultures grown on LB broth at 10,000 x* g* for 20 min were analyzed by sodium dodecyl sulfate-polyacrylamide gel electrophoresis. After electrophoresis, the gels were stained with Coomassie brilliant blue. Exoprotease activities of culture supernatants from psychrotrophic strains were also analyzed by SDS-PAGE with 0.2% (w/v) azocasein incorporated into the gel matrix (12% polyacrylamide) as described before [4].

Lipolytic activity was determined by streaking the strains on medium 884 (Tween 80-Agar). Lipolytic activity on* p*-nitrophenyl palmitate was also investigated using 100 *μ*L bacterial supernatant from overnight cultures in LB or TYEP [4, 8].

### 2.4. Detection of AHL by Cross-Streaking

AHL-production was investigated by cross-streaking psychrotrophic strains that were grown overnight on LB agar plates against monitor strains as previously performed [9].

### 2.5. Extraction of AHL from Supernatants

An inoculum of 10^4^ CFU/mL of psychrotrophic strains was added to 250 mL of LB. Samples were incubated with aeration at 30°C for 20 h or until the population reaches 10^9^ CFU/mL. Then, the cells were harvested by centrifugation at 10,000 x* g* for 20 min, and cell-free supernatants were used for AHL extraction [13].

### 2.6. Detection of AHL by Thin-Layer Chromatography (TLC)

Twenty *μ*L aliquots of extracts were loaded onto TLC plates as described in Pinto [9]. A volume of 150 mL of soft agar at 42°C was mixed with 30 mL of the monitor strain* E. coli* MT102 (pSB403) or* C. violaceum* CV026. The added soft agar of the appropriated monitor strain was dispensed onto TLC plate receiving a 2 to 3 mm thick layer. After 20 min, the plate was put in an airproof box with a wet paper inside and incubated overnight at 30°C.

The documentation was dependent on the monitor strain used. For* C. violaceum* CV026, the material was incubated until 48 h and the signal molecules could be identified by forming violet pigmented spots. When* E. coli* MT102 pSB403 was the monitor, the material was incubated overnight at 30°C and put into a dark box and the bioluminescence was detected with a highly sensitive photon-counting camera (C2400-40; Hamamatsu Photonics Herrsching, Germany).

### 2.7. LC-MS Analysis of AHL Extracts from Bacterial Supernatants

One hundred and twenty *μ*L of dichloromethane extracts from 400 mL of culture supernatant in LB medium were evaporated under a gentle stream of nitrogen. The residue was redissolved in 120 *μ*L of aqueous methanol 60% (v/v) and separated by reversed-phase LC-MS (C18 column, Grom-Sil 120 ODS-4 HE, 4.6 x 250 mm, Stagroma, Germany) under the following conditions: flow rate 1 mL/min; solvent A: UV-treated H_2_O and 0.1% formic acid; solvent B: acetonitrile (ACN) and 0.1% formic acid, and it was subsequently analyzed by mass spectrometry (LCQ Duo Mass Spectrometer, Thermoquest, Finnigan) equipped with an electrospray source. The following gradient was applied: solvent B from 25% ACN to 100% in 20 min, isocratic, 5 min.

### 2.8. DNA Manipulations, PCR Reactions, and Sequencing of* hal*I and* hal*R Genes

Cloning, restriction enzyme analysis, and transformation of* E. coli* were performed using established procedures [14]. To amplify the AHL synthase gene (*halI*) and the AHL receptor gene* halR* by PCR, the reaction consisted of 25 mM MgCl_2_, 5.0 *μ*L of 10X buffer Ex* Taq*, 25 mM deoxynucleotide triphosphates (dNTPs), 25 *μ*M of each primer, 1 U Ex* Taq* DNA polymerase, and 40 ng of DNA from* H. alvei *068 in a final volume of 50 *μ*L. Primers based on the sequences of* halI* and* halR *genes (GenBank accession number AF503776) of* H. alvei* were constructed (see Table 2) and synthesized by Microsynth (Zürich, Switzerland). PCR reactions were carried out in a T3 thermocycler (Biometra®, Biolabo Scientific Instruments, Zürich, Switzerland).

The M13 Universal and Reverse Primers were used to sequence* halI* and* halR* genes cloned into pCR2.1-TOPO.

### 2.9. Cloning and Heterologous Expression of AHL Synthase (HalI) of* H. alvei* 068 in pQE-30Xa

Once the complete sequence of the* halI* gene was obtained, primers were designed to amplify the* halI* open reading frame (ORF) by PCR using the bacterial genomic DNA as a template and TaKaRa Ex Taq polymerase (see Table 2). Primers generated* Bam*HI and* Sac*I sites at the 5' and 3' ends of the amplicons, respectively (see Table 2). The DNA amplicon, 660 bp, containing the* halI* structural gene was digested with* Bam*HI and* Sac*I and ligated into vector pQE-30Xa (Qiagen), previously cut with the same restriction enzymes. This plasmid harboring the ORF of* halI *inserted downstream of the T5 promoter was named pQE-30Xa-halI068. Plasmid pQE-30Xa-halI068 was transformed into the expression strain* E. coli* XL1-Blue.

For overproduction of HalI,* E. coli* XL1-Blue cells carrying pQE-30Xa-halI068 were grown in dYT medium (tryptone 1.6%, yeast extract 1.0%, NaCl 0.5%, and glucose 0.2%) containing ampicillin (100 *μ*g mL^−1^) at 37°C under vigorous shaking. At an optical density at 600 nm of 0.5, isopropyl-*β*-D-thiogalactopyranoside (IPTG) was added to the culture to a final concentration of 1 mM. After 5 h incubation at 37°C, the cells were collected by centrifugation at 10,000 x* g* for 30 min and resuspended in 50 mM Tris-HCl (pH 8.0). Then, 3 *μ*L of cell suspension were loaded onto SDS-PAGE (15%) in order to detect HalI overexpression.

### 2.10. Detection, Extraction, and Characterization of AHL Produced by HalI

AHL-production was investigated by cross-streaking* E. coli* XL1-Blue pQE-30Xa-halI068 that was grown overnight on dYT agar plates supplemented with 1 mM IPTG against* E. coli* pSB403 or* C. violaceum* CV026.

In order to extract AHL, 10^4^ CFU/mL of* E. coli* XL1-Blue pQE-30Xa-halI068 were inoculated in 250 mL of dYT. At an optical density at 600 nm of 0.5, IPTG was added to the culture to a final concentration of 1 mM. The samples were incubated with aeration at 30°C up to 48 hours. Then, the cells were harvested by centrifugation at 10,000 x* g* for 20 min, and cell-free supernatants were used to extract AHL [13].

Detection of AHL by Thin-Layer Chromatography (TLC) was done as described in item 2.6, as well as the chemical characterization by LC-MS analysis of AHL molecules present in the extracts from bacterial supernatants (item 2.7).

### 2.11. Autoinducer 2 Production in Psychrotrophic Strains

Psychrotrophic strains were grown overnight with aeration at 30°C on LB medium. Cell-free culture supernatants were prepared by removing the cells from the growth medium by centrifugation at 10,000 x* g* for 20 min. The cleared culture supernatants were passed through 0.2 *μ*m filters and stored at -20°C. As a positive control,* V. harveyi* BB120 was grown overnight at 30°C with aeration in AB medium [15], which is optimal for* Vibrio *species and commonly used in AI-2 assays. It is composed of NaCl 0.30 M, MgSO_4_ 0.05 M, vitamin-free casamino acids 0.2 %, and pH 7.5 (adjusted with KOH). The medium was sterilized and cooled, and 10 ml of sterile 1 M potassium phosphate (pH 7.0), 10 mL of 0.1 M L-arginine, 20 mL of glycerol, 1 mL of 10 *μ*g/mL riboflavin, and 1 mL of 1 mg/mL thiamine per L were added. Cell-free culture supernatants from* V. harveyi* BB120 were prepared from overnight culture by centrifugation at 10,000 x* g* for 20 min. Aliquots of 10 *μ*L of cell-free culture fluids were added to 96-well microtiter plates. The monitor strain,* V. harveyi* BB170, was grown with aeration for 16 h at 30°C in AB medium and diluted as 1:5.000 into fresh AB medium. Aliquots of 90 *μ*L of diluted cells were added to wells containing the 10 *μ*L psychrotrophic strains cell-free culture fluids. Positive control wells contained 10 *μ*L of cell-free culture fluid from* V. harveyi* BB120 and negative control wells contained 10 *μ*L of sterile growth medium (LB or AB). Microtiter dishes were shaken in a rotary shaker at 175 RPM at 30°C. Bioluminescence was measured using the KC4 (Bio-Tek Instruments, Highland Park, Box 998, Vermont, USA).

## 3. Results and Discussion

### 3.1. Identity of the Psychrotrophic Strains Isolated from Cooled Raw Milk

The identity of the strains characterized in this study is shown in Table 3. We were unable to show the species level for isolate 039 (see Table 3), identified only as* Pantoea* sp. by the 16S rDNA sequencing. The biochemical characterization by using the API ID32E was inconclusive for isolates 039 and 059. Pinto [9] showed that these psychrotrophic isolates were able to induce quorum sensing biosensor strains, but further characterization of the quorum sensing signal molecules repertoire and their spoilage potential was not performed. Additionally, we decided to characterize these isolates because they belong to the Enterobacteriaceae family, as well as their prevalence in Brazilian milk [24].

### 3.2. Spoilage Potential and Production of Exoenzymes

**Figure 1:**
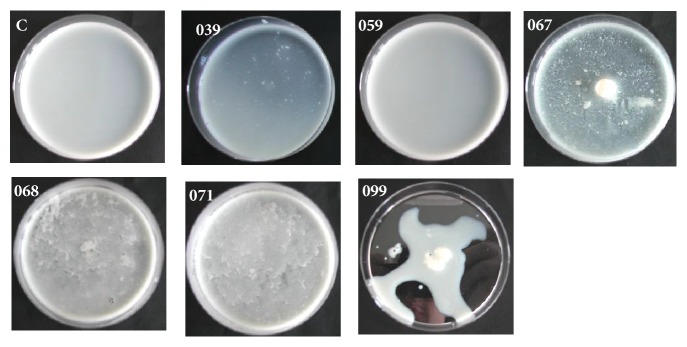
Spoilage ability of psychrotrophic strains inoculated in reconstituted skim milk powder, 12% (w/v). (C) Negative control, milk sample not inoculated, (039)* Pantoea *sp., (059)* H. alvei*, (067)* Enterobacter* sp., (068)* H. alvei*, (071)* H. alvei*, and (099)* A. hydrophila*.

The strains evaluated in this study showed different abilities to spoil milk samples as shown in Figure 1. As it can be observed,* A. hydrophila* 099 was the most deteriorative, whereas* Pantoea *sp. 039 had lower ability to spoil milk, as well as* H. alvei* 059 (see Figure 1).

Bacterial spoilage causes significant economic losses for the dairy industry, and different psychrotrophic strains can show different spoilage potentials as confirmed in this and many other studies [1, 2, 4]. Additionally, many works have shown the spoilage potential of* Pseudomonas* spp. isolated from milk samples, but only a few have investigated the role that other species have on milk deterioration [2, 25], which explains our interest in characterizing isolates that belong to the Enterobacteriaceae family.

The proteolytic activity of some extracellular enzymes of* A. hydrophila* has been recognized and it is considered to play a major role in the virulence and pathogenicity of this bacterium [26]. Besides, Vivas [27] showed that this microorganism can produce and secrete proteases able to cleave milk proteins. According to Cousin [28], proteases produced by* Aeromonas* are able to degrade *α*-, *β*-, *κ*-, and *γ*-casein as well as the whey proteins. Khajanchi [29] have shown the involvement of quorum sensing in the control of protease production and* in vivo* virulence of a strain of clinical significance of* A. hydrophila.*

All three strains of* H. alvei* presented different spoilage potentials underscoring the genetic variability of these isolates (see Figure 1). According to Bruhn [30],* H. alvei* was the dominant member of Enterobacteriaceae in vacuum-packed meat, possibly inducing food quality-relevant phenotypes in other bacterial species in the same environment. In addition to the possibility of inducing phenotypes in other bacterial species, two of our strains were able to spoil milk samples (see Figure 1).

Although* Enterobacter* sp. is normally isolated from raw and pasteurized milk and butter [28], it is not considered a potent dairy spoiler bacterium. However, in this study, it was verified that strain 067 presented a potential to spoil reconstituted skim milk samples (see Figure 1).

**Figure 2:**
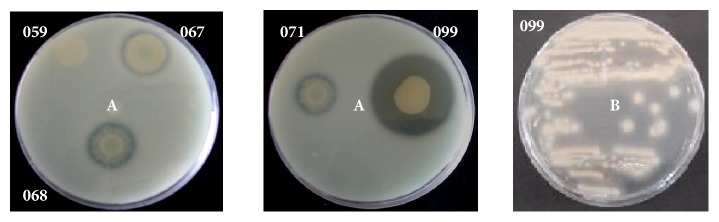
(A) Proteolytic activity on LB agar supplemented with 2% (w/v) skim milk powder. Clearing zones are indicative of protease activity. (059)* H. alvei*, (067)* Enterobacter* sp., (068)* H. alvei*, (071)* H. alvei*, and (099)* A. hydrophila*. (B) Lipolytic activity after growth of* A. hydrophila* 099 on Tween 80-Agar for 48 h at 30°C. Precipitation zones are indicative of lipase activity.

In order to confirm the proteolytic nature of the bacterial isolates, we streaked them onto LB agar plates supplemented with 2% skim milk powder and it was once again verified that they had different abilities to produce proteolytic enzymes able to cleave casein (see Figure 2). These results confirmed that* A. hydrophila* produced the highest amount of exoproteases compared to the other strains judging by the diameter of the clearing zone (see Figure 2).* H. alvei* 059 and* Pantoea* sp. 039 were unable to hydrolyze casein in this assay, confirming our previous results (see Figure 1).

We then sought to verify the extracellular proteolytic activity in supernatant of cultures grown on LB and TYEP broth media. However, strains 039, 059, 067, 068, and 071 did not produce detectable levels in this assay. It is likely that the azocasein is not a good substrate for determination of proteolytic activity produced by these strains or that they do not produce these enzymes on the broths chosen for the assays. Their ability to spoil milk shown in Figures 1 and 2 should not be neglected.

**Figure 3:**
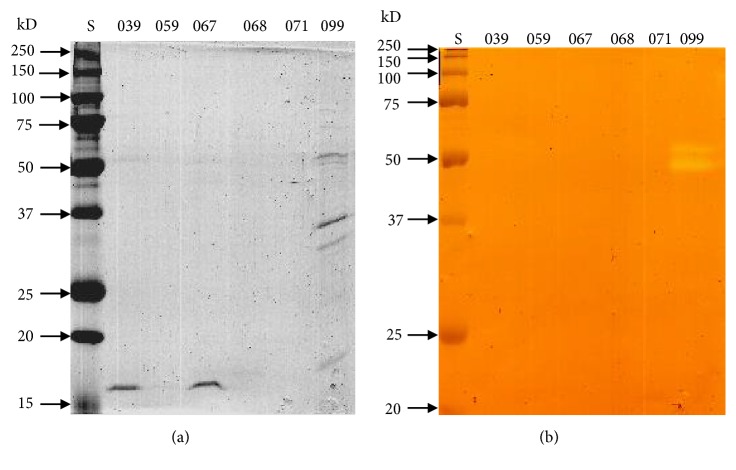
SDS-PAGE (a) and zymogram azocasein (b) gel (12%) showing protease production by psychrotrophic strains after growth in LB medium. Lines: (S) standards of molar mass, (039)* Pantoea *sp. (059),* H. alvei*, (067)* Enterobacter* sp., (068)* H. alvei*, (071)* H. alvei*, and (099)* A. hydrophila*.

In contrast, strain 099 showed proteolytic activity of 0.131 units/h/*μ*g of protein in TYEP. Many extracellular proteins were observed in the supernatant obtained from* A. hydrophila* 099 (see Figure 3(a)), and two of them had proteolytic activity on SDS-PAGE supplemented with 2% azocasein (see Figure 3(b)). Production of both serine protease and metalloprotease activities in* A. hydrophila* is under the control of quorum sensing mechanism [31]. However, Ponce-Rossi [32] challenged this idea when using a quorum sensing defective strain of* A. hydrophila *that continued to produce proteases, although at low levels.

No proteolytic activity was observed in the supernatant of the other strains grown in LB broth (see Figure 3(b)). Since previous data show that isolates 067, 068, and 071 present proteolytic activity in milk (see Figures 1 and 2), the effect of medium components should be evaluated in a future study to better clarify the involvement of environmental parameters on the protease production by these strains.

### 3.3. Lipase

In this study, only strain 099 showed lipolytic activity of 1.104 units/h/*μ*g protein in TYEP medium. This activity was confirmed on Tween 80-Agar (see Figure 2B). The other strains did not present detectable lipase levels on this medium. According to Brumlik and Buckley [33], among extracellular enzymes released by* A. hydrophila,* a glycerophospholipid-cholesterol acyltransferase (GCAT) has been described and characterized. Lipolytic activity of other* A. hydrophila *strains has also been verified by Ponce-Rossi [32].

### 3.4. Detection of AHL Signaling Molecules

The tested psychrotrophic proteolytic strains induced many of the biosensor strains, as shown in Table 4. As we used a range of different AHL monitor systems, it is possible that we have covered a wide range of known AHLs. Other members of Enterobacteriaceae isolated from food sources have been shown to produce signaling AHL molecules [9, 20].

Strains 059, 068, and 071 of* H. alvei* produced higher amounts of AHL than the others once they were able to strongly induce the monitor strains (see Table 4). Pinto [9] demonstrated that AHL-production is common among psychrotrophic bacteria isolated from milk and suggested that quorum sensing may play an important role in the spoilage of this product.

### 3.5. Characterization of AHL Molecules by TLC Analyses

**Figure 4:**
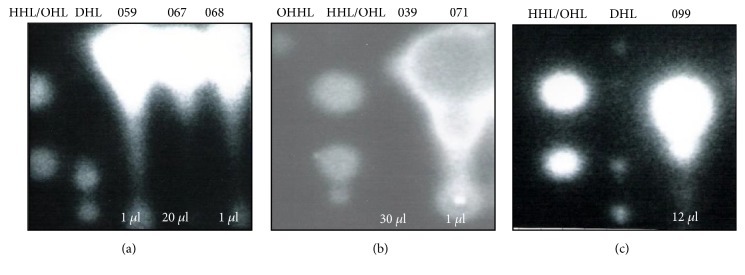
Representative thin-layer chromatograms of the signal molecules present in cell-free supernatants of Enterobacteriaceae strains isolated from cooled raw milk and cultivated in LB medium. The spots were detected with* E. coli* pSB403 reporter strain. Standards: N-(hexanoyl)-DL-homoserine lactone (HHL); N-(octanoyl)-L-homoserine lactone (OHL); N-(dodecanoyl)-L-homoserine lactone (DHL); N-(3-oxohexanoyl)-L-homoserine lactone (OHHL). (059)* H. alvei*, (067)* Enterobacter* sp., (068)* H. alvei*, (039)* Pantoea* sp., (071)* H. alvei*, and (099)* A. hydrophila*.

**Figure 5:**
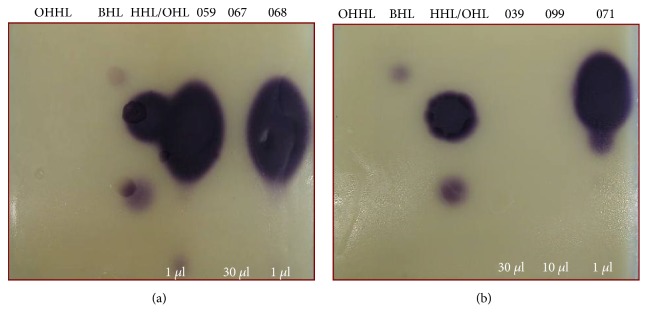
Representative thin-layer chromatograms of the signal molecules present in cell-free supernatants of Enterobacteriaceae isolated from cooled raw milk and cultivated in LB medium. The spots were detected with* C. violaceum* CV026 reporter strain. Standards: N-(3-oxohexanoyl)-L-homoserine lactone (OHHL); N-(butanoyl)-L-homoserine lactone (BHL); N-(octanoyl)-L-homoserine lactone (OHL); N-(hexanoyl)-DL-homoserine lactone (HHL). (059)* H. alvei*, (067)* Enterobacter* sp., (068)* H. alvei*, (039)* Pantoea* sp., (099)* A. hydrophila*, and (071)* H. alvei*.

The results from the TLC plates confirmed those obtained on the cross-streak experiment. Accordingly, no response to the extract prepared from* Pantoea *sp. 039 was observed on the TLCs revealed with* E. coli* pSB403 or* C. violaceum *CV026 (see Figures 4(b) and 5(b)). Different amounts of AHLs were detected on the TLC plates for the other strains (see Figures 4 and 5). For instance, the strains* Enterobacter* 067 and* A. hydrophila* 099 produced less amount of AHL compared to* H. alvei* 059, 068, and 071, since it was necessary to load higher volumes of AHL extracts onto the TLC plate in order to detect production of bioluminescence by* E. coli* pSB403 (see Figures 4(a) and 4(c)). Besides, the extracts obtained from* Enterobacter* 067 and* A. hydrophila* 099 were not able to induce* C. violaceum* CV026 (see Figures 5(a) and 5(b)).

Interestingly, we observed degradation products of N-(dodecanoyl)-L-homoserine lactone (DHL) on TLC plates (see Figures 4(a) and 4(c)) which suggests high sensitivity of this AHL molecule to the experimental conditions.* C. violaceum* CV026 was unable to detect N-(3-oxohexanoyl)-L-homoserine lactone (OHHL) (see Figure 5), highlighting the importance of multiple AHL sensors systems to detect a broader range of AHL molecules.

### 3.6. Characterization of AHL Molecules by Liquid Chromatography-Mass Spectrometry (LC-MS)

Isolates 059, 068, 071, and 099 produced different AHL molecules (see Table 5). However, it was not possible to detect any AHL molecule from the AHL extract of* Enterobacter *sp. 067, even though it induced the biosensors in the cross-streak assay and on the TLCs.

*H. alvei* isolates 059, 068, and 071 produced 3-oxo-C6-HSL, C6-HSL, and 3-oxo-C8-HSL, whereas C8-HSL was produced by* H. alvei* 059 and 071 (see Table 5). In the experimental conditions adopted, 3-oxo-C6-HSL was the main AHL produced by* H. alvei* strains. This result agrees with those from Bruhn [30] who verified that this same HSL was predominant among four AHLs produced by* H. alvei* isolated from vacuum-packed meat. However, Hou [34] detected C4-HSL, C6-HSL, and 3-oxo-C8-HSL in culture supernatants of* H. alvei* H4 isolated from spoiled sea cucumber, revealing the diversity of signaling molecules in* H. alvei* species.

On the other hand,* A. hydrophila* 099 produced C4-HSL and C6-HSL (see Table 5), confirming the results of Swift [35] and Nagar [36] who demonstrated that* A. hydrophila* produces C4-HSL as the major AHL molecule. Quorum sensing in this pathogen has been associated with regulation of biofilm development [37, 38] and exoprotease production [31].

*H. alvei* produced a molecule that presented mass spectrum similar to 3-hydroxy-C4-HSL and 3-hydroxy-C12-HSL, whereas* A. hydrophila* 099 probably produced C5-HSL (see Table 5). However, the identity of these molecules was not confirmed since standards of these compounds were not available to determine their mass spectrum and retention time.

Characterization of different AHLs by LC-MS reinforces the data obtained in the cross-streak experiments in which different biosensor strains were induced in this study. Each AHL biosensor relies on a specific LuxR homologue, thus displaying specificity towards its cognate AHL and in some cases to closely related AHLs [39].

### 3.7. *halI *and* halR *Gene Characterization by PCR and Sequencing

**Figure 6:**
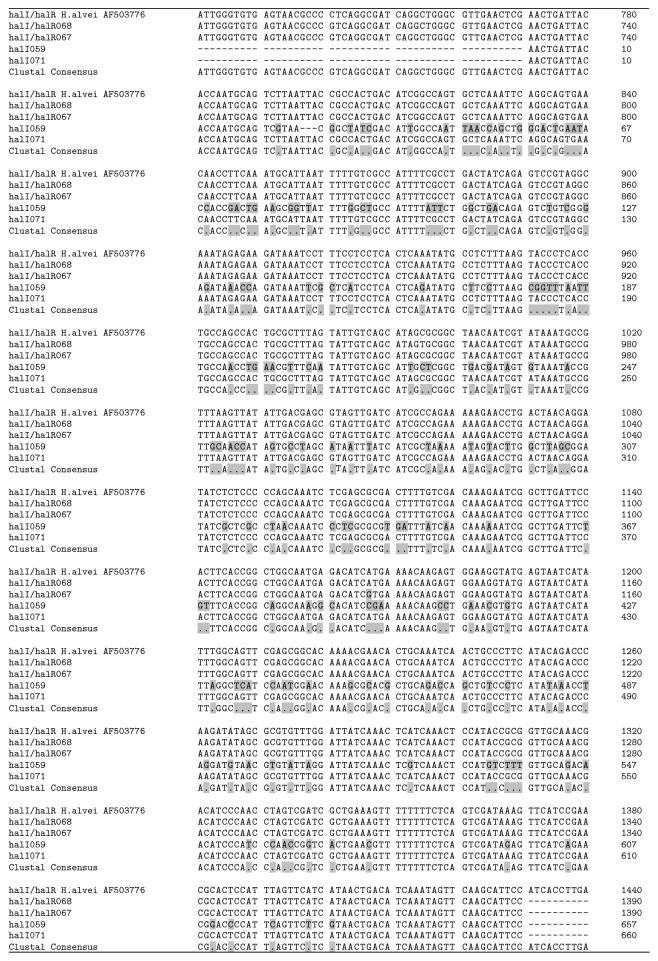
Multiple sequence alignment of* halI *gene of* H. alvei* 059, 068, 071, and* Enterobacter* sp. 067 (this study) with* halI* gene of* H. alvei* (Genbank accession number AF503776). The differences of identity are indicated by gray shading.

Amplified products of expected size, 660 bp or 751 bp, were obtained for* halI* and* halR* genes, respectively. Additionally, the* halI* gene was detected in all strains of* H. alvei* used in this study as well as in* Enterobacter* sp. 067. The* hal*I gene of* H. alvei *068 and 071 showed 99% identity with one another. The same result was observed when the* halI* and* halR* genes of* H. alvei *068 were compared to* halI* and* halR* genes of* Enterobacter *sp. 067 (see Figure 6). However, when sequences of* hal*I gene of* H. alvei *068 and* H. alvei *059 were aligned, they showed 75% identity with each other. Differences in the sequences of these genes may account for the differences in the phenotypic tests we have observed, including the different AHL profiles.

### 3.8. Sequencing and Overexpression of* halI* in* E. coli* XL1-Blue

In order to confirm which AHL molecules are synthesized by HalI,* halI* gene from* H. alvei* 068 was sequenced, cloned, and overexpressed in* E. coli* XL1-Blue. This gene comprised 660 bp and coded for a protein of 216 amino acids. Based on electrophoretic mobility, the molecular mass of this enzyme was determined to be approximately 16 kDa and was soluble in the conditions used in this study. Size chain of HalI was in agreement with LuxI-type proteins that usually contain between 194 and 226 amino acids [40].

### 3.9. HalI Produces AHL Molecules Ectopically in* E. coli*

**Figure 7:**
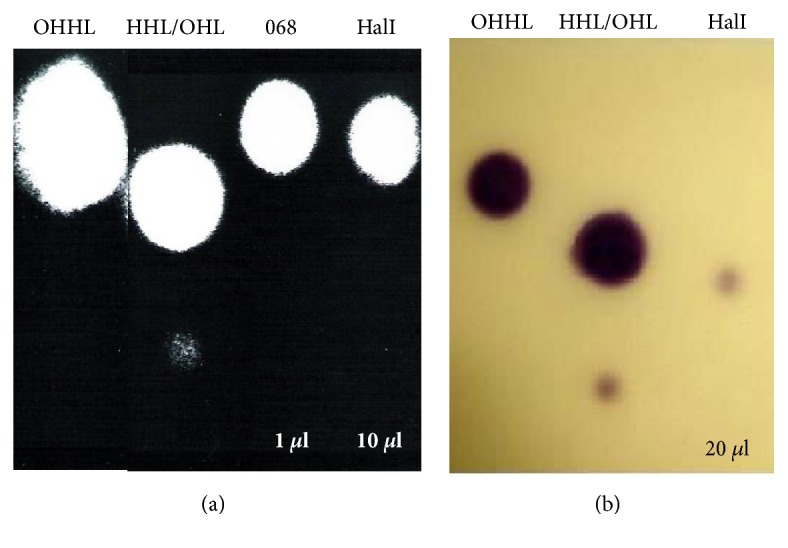
(a) A representative thin-layer chromatogram of HalI expression in* E. coli* XL1-Blue cultured in LB medium. The spots were detected with* E. coli* pSB403 reporter strain. Standards: N-(3-oxohexanoyl)-L-homoserine lactone (OHHL); N-(hexanoyl)-L-homoserine lactone (HHL); N-(octanoyl)-L-homoserine lactone (OHL); (068)* H. alvei *wild type; AHL extract diluted 50 times in ethyl acetate; (HalI)* E. coli* XL1-Blue harboring pQE-30Xa-halI. (b) A representative thin-layer chromatogram of HalI expression in* E. coli* XL1-Blue cultured in LB medium. Spots were detected with* C. violaceum *CV026 reporter strain. Standards: N-(3-oxohexanoyl)-L-homoserine lactone (OHHL); N-(hexanoyl)-L-homoserine lactone (HHL); N-(octanoyl)-L-homoserine lactone (OHL); (HalI)* E. coli* XL1-Blue harboring pQE-30Xa-halI.

Cross-streak between* E. coli* XL1-Blue harboring pQE-30Xa-*halI* and biosensors* E. coli* pSB403 and* C. violaceum* CV026 was performed confirming induction of both monitor strains, which indicates successful expression and activity of HalI in* E. coli*. We then analyzed supernatant extracts of* E. coli* XL1-Blue harboring pQE-30Xa-*halI* cultured in LB medium confirming the induction of* E. coli* pSB403 and* C. violaceum* CV026 on the TLC assays (see Figure 7). The spots observed on TLC where* E. coli* pSB403 was used as biosensor presented the same retention factor (rf) of 3-oxo-C6-HSL (see Figure 7), while a smaller spot close to C6-HSL was detected on TLC developed with* C. violaceum* CV026 (see Figure 7). These results indicated that* halI* gene codes for an enzyme able to synthesize 3-oxo-C6-HSL and C6-HSL.

**Figure 8:**
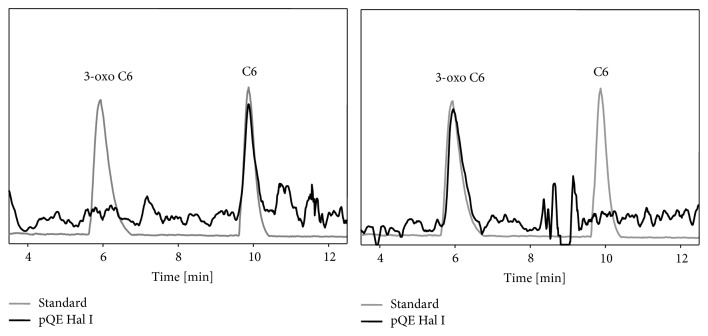
High-performance liquid chromatography-positive electrospray ionization (ESI^+^-) MS chromatogram showing the mass spectra for the signal molecules present in cell-free supernatant of* E. coli* XL1-Blue pQE-30Xa-halI. Signal molecule extract was obtained from overnight cell-free culture supernatant in LB minimal medium.

Besides characterizing the products of HalI expression by TLC, we have also analyzed the extracts by LC-MS confirming the production of C6-HSL and 3-oxo-C6-HSL (see Figure 8). Interestingly, the parental* H. alvei *strain produced additional molecules as shown on Table 5. It is not clear why when* halI* was expressed in* E. coli, *it directed the synthesis of only two AHLs. We envision three possible scenarios that could explain these results: one in which the diversity of substrates (acyl-carrier proteins) was not present or had insufficient concentration in* E. coli;* also HalI could have produced levels of other AHLs that were not detected in our assays; and lastly an additional synthase is present in* H. alvei*.* P. aeruginosa* is a good example of a bacterium that contains two synthases (LasI and RhlI), both of which direct the synthesis of different AHL molecules and coordinate a complex quorum sensing cascade [6].

### 3.10. Detection of Autoinducer 2

Sterilized supernatant of overnight culture of* A. hydrophila* 099 in LB broth was able to induce bioluminescence production of AI-2 monitor strain* V. harveyi *BB170 (see Table 6), confirming the results of Jahid [41]. Additionally,* H. alvei *isolates 059, 068, and 071 produced AI-2 under our experimental conditions. To our knowledge this is the first report to show AI-2 production by* H. alvei* strains. In contrast,* Pantoea *sp. 039 and* Enterobacter *067 did not produce any detectable levels of AI-2. It is noteworthy that the majority of the evaluated strains in this study are able to communicate via two different quorum sensing systems, underscoring the importance of these signaling mechanisms in the food related environments.

## 4. Conclusions

*A*. h*ydrophila *099 presented the highest potential to spoil milk followed by* H. alvei *068 and 071 and* Enterobacter *sp. 067 strains. A diverse array of AHL molecules was produced by these strains, as confirmed by different assays. The most common AHLs produced by* H. alvei* strains were C6-HSL and 3-oxo-C6-HSL, which was confirmed by ectopic expression of HalI synthase on an* E. coli *host*. A. hydrophila *099 strongly induced the AI-2 monitor strain* V. harveyi *BB170. Taken together, these results highlight the spoilage potential of Enterobacteriaceae strains isolated from cooled raw milk and a rich array of signaling molecules produced by these microorganisms which likely influence many bacterial traits in the food environment. Quorum sensing inhibition strategies are suggested as potential barriers to milk spoilage and increased milk safety. We propose studies that use quorum sensing inhibition strategies as additional barriers in the milk processing environment.

## Figures and Tables

**Table 1:** Bacterial strains and plasmids used in this study.

**Strain**	**Plasmid**	**Description**	**Reference or source**
*Aeromonas hydrophila *099		Wild type, psychrotrophic isolated from cooled raw milk	[10]
*Agrobacterium tumefaciens* A136	pCF373, pCF218, Tc^r^, Spc^r^	Monitor strain: detects AHL with 3-oxo, 3-hydroxy, and 3-unsubstituted side chain	[16]
*Agrobacterium tumefaciens* NTL4	pZLR4, Gm^r^	Monitor strain: detects AHL with 3-oxo, 3-hydroxy, and 3-unsubstituted side chain	[17]
*Burkholderia cepacia* H111		Positive control in the cross-streak to *E. coli* pSB403, and *P. putida* F117 pAS-C8	[18]
*Burkholderia vietnamiensis*		Positive control in the cross-streak to* P. putida* F117 pKR-C12	[19]
*Chromobacterium violaceum* CV026		Monitor strain: detects AHL compounds with unsubstituted side chains from C4 to C8 in length.	[20]
*Enterobacter* sp. 067		Wild type, psychrotrophic isolated from cooled raw milk	[10]
*Escherichia coli* MT102	pSB403, Tc^r^	Monitor strain: exhibits the highest sensitivity for 3-oxo-C6-HSL. However, several other AHL molecules are detected by this sensor	[21]
*Escherichia coli *XL1-Blue	pQE30-Xa	Cloning and subcloning host. *sup*E44, *hsd*R17, *end*A1, *rec*A1, g*yr*A96, *thi*1, *rel*A1, *lac*- F′[*pro*AB+, *lacIq*, *lac*Z∆M15, Tn10 (*tet*F)]	[22]
*Escherichia coli *XL1-Blue	pQE30-Xa-halI068	It expresses AHL synthase, HalI, from *H. alvei* 068	This study
*Hafnia alvei* 059		Wild type, psychrotrophic isolated from cooled raw milk	[10]
*Hafnia alvei* 068		Wild type, psychrotrophic isolated from cooled raw milk	[10]
*Hafnia alvei* 071		Wild type, psychrotrophic isolated from cooled raw milk	[10]
*Pantoea *sp. 039		Wild type, psychrotrophic isolated from cooled raw milk previously identified as *Serratia liquefaciens*	[10]
*Pseudomonas aeruginosa* PAO1		Positive control in the cross-streak to *C. violaceum* CV026, *A. tumefaciens* NTL4, and *A. tumefaciens* A136	Laboratory of Microbiology, University of Zürich
*Pseudomonas putida* F117	pAS-C8, Gm^r^	Monitor strain: exhibits the highest sensitivity for OHL	[19]
*Pseudomonas putida* F117	pKR-C12, Gm^r^	Monitor strain: it detects 3-oxo-C12- and 3-oxo-C10-HSL	[19]
*Vibrio harveyi *BB120		Positive control: AI2 producer	[23]
*Vibrio harveyi *BB170		Monitor strain: detects AI2	[23]

**Table 2:** Primers used to amplify *hal*I and *hal*R genes by PCR.

Primer	Sequence (5'-3')	Application
halI-F	AACTGATTACACCAATGCAGT	Amplification of *hal*I
halI-R	GGAATGCTTGAACTATTTGATG	Amplification of *hal*I
halI-bam	ATTGGATCCTACACCAATGCAGTCTTAATT	Amplification of *hal*I gene and preparation for cloning in pQE-30Xa
halI-sac	ATTGAGCTCATGCTTGAACTATTTGATGTC	Amplification of *hal*I gene and preparation for cloning in pQE-30Xa
halR-F	CTT CAG GGA TGC CAT ATG TTT	Amplification of *hal*R
halR-R	ACT GCA TTG GTG TAA TCA GTT	Amplification of *hal*R

The introduced restriction sites for BamHI and SacI are underlined.

**Table 3:** Identification of Enterobacteriaceae isolated from cooled raw milk.

Isolate	API ID32E	rDNA 16S
039	Nd*∗*	*Pantoea *sp.
059	Nd	*Hafnia alvei*
067	*Enterobacter cloacae*	*Enterobacter* sp.
068	*Hafnia alvei*	*Hafnia alvei*
071	*Hafnia alvei*	*Hafnia alvei*
099	*Aeromonas hydrophila*	*Aeromonas hydrophila*

*∗*Nd: not determined (inconclusive results).

**Table 4:** Activation of the AHL monitor strains in cross-streak experiments.

Isolate and controls	Monitor strains					
	CV 026	pSB403	F117 (pAS-C8)	F117 (pKR-C12)	A 136	NTL4
*Pantoea* sp. 039	-	-	-	-	+	++
*H. alvei* 059	+++	+++	++	-	+++	+++
*Enterobacter *sp. 067	+	++	-	-	+	++
*H. alvei* 068	+++	+++	++	-	+++	+++
*H. alvei* 071	+++	+++	+	-	+++	+++
*A. hydrophila *099	++	++	+	-	+	+++
*B. cepacia* H111	Nd	+++	+++	Nd	Nd	Nd
*B. vietnamiensis*	Nd	Nd	Nd	+++	Nd	Nd
*P. aeruginosa* PAO1	+++	Nd	Nd	Nd	+++	+++

The six monitor strains were cross-streaked against different psychrotrophic strains on LB agar plates. Following up to 48 hours of incubation at 30°C, the production of violacein by *C. violaceum* CV026, bioluminescence by *E. coli* pSB403, green fluorescent protein gfp (ASV) by *P. putida* F117, and *β*-galactosidase activity by *A. tumefaciens* A136 and NTL4 was visualized as described in the Material and Methods. Levels of activation are indicated as follows: +++, strong activation, diffusion of AHL of > 1 cm; ++, activation, diffusion of AHL of 0.5 to 1 cm; +, weak activation, diffusion of AHL of < 0.5 cm; -, no detectable activation. Nd: not determined.

**Table 5:** Summary of identification by high-performance liquid chromatography positive electrospray ionization (ESI^+^-) MS of AHLs produced by *H. alvei* 059, 068, and 071, *Enterobacter* sp. 067, and *A. hydrophila* 099.

Standard	[M+H]^+1^	Retention time of AHL molecules [min]						
		Standard mix Calibration^1^	067	068	071	099	Standard mix Calibration	059
C4-HSL	172	5.5	-^3^	-	-	5.8	4.29	-
3-hydroxy-C4-HSL	188	Nd^2^	-	4.6	-	-	Nd	-
C5-HSL	186	Nd	-	-	-	7.8	Nd	-
3-Oxo-C6-HSL	214	6.6	-	6.5	6.6	-	4.57	4.55
C6-HSL	200	10.9	-	10.9	11.0	10.9	9.69	9.45
3-Oxo-C8-HSL	242	12.5	-	12.5	12.5	-	11.18	11.16
C8-HSL	228	15.3	-	-	15.4	-	14.26	14.2
3- hydroxy-C12-HSL	300	Nd	-	17.2	17.2	-	Nd	-
C10-HSL	256	18.6	-	-	-	-	17.59	-
3-Oxo-C12-HSL	298	19.0	-	-	-	-	Nd	-

^1^[M+H]^+^, mass to charge rate. ^2^Nd, not determined. ^3^Nothing found.

**Table 6:** Detection of autoinducer 2 in supernatant of LB medium inoculated with psychrotrophic strains.

Strains and medium	Luminescence at 175 nm*∗*
*Pantoea *sp. 039	1973 ± 345
*H. alvei *059	2948 ± 810
*Enterobacter *sp. 067	2087 ± 439
*H. alvei *068	2899 ± 606
*H. alvei *071	3708 ± 687
*A. hydrophila *099	12903 ± 192
*V. harveyi* BB120	4478 ± 390
LB medium	2299 ± 384

^*∗*^Average and standard deviation of data are shown. n: number of repetitions equal to 8.

## Data Availability

The data used to support the findings of this study are available from the corresponding author upon request.
